# Human and Environmental Impacts on River Sediment Microbial Communities

**DOI:** 10.1371/journal.pone.0097435

**Published:** 2014-05-19

**Authors:** Sean M. Gibbons, Edwin Jones, Angelita Bearquiver, Frederick Blackwolf, Wayne Roundstone, Nicole Scott, Jeff Hooker, Robert Madsen, Maureen L. Coleman, Jack A. Gilbert

**Affiliations:** 1 Graduate Program in Biophysical Sciences, University of Chicago, Chicago, Illinois, United States of America; 2 Institute for Genomics and Systems Biology, Argonne National Laboratory, Lemont, Illinois, United States of America; 3 Biology Department, Chief Dull Knife College, Lame Deer, Montana, United States of America; 4 Department of the Geophysical Sciences, University of Chicago, Chicago, Illinois, United States of America; 5 Department of Ecology and Evolution, University of Chicago, Chicago, Illinois, United States of America; U. S. Salinity Lab, United States of America

## Abstract

Sediment microbial communities are responsible for a majority of the metabolic activity in river and stream ecosystems. Understanding the dynamics in community structure and function across freshwater environments will help us to predict how these ecosystems will change in response to human land-use practices. Here we present a spatiotemporal study of sediments in the Tongue River (Montana, USA), comprising six sites along 134 km of river sampled in both spring and fall for two years. Sequencing of 16S rRNA amplicons and shotgun metagenomes revealed that these sediments are the richest (∼65,000 microbial ‘species’ identified) and most novel (93% of OTUs do not match known microbial diversity) ecosystems analyzed by the Earth Microbiome Project to date, and display more functional diversity than was detected in a recent review of global soil metagenomes. Community structure and functional potential have been significantly altered by anthropogenic drivers, including increased pathogenicity and antibiotic metabolism markers near towns and metabolic signatures of coal and coalbed methane extraction byproducts. The core (OTUs shared across all samples) and the overall microbial community exhibited highly similar structure, and phylogeny was weakly coupled with functional potential. Together, these results suggest that microbial community structure is shaped by environmental drivers and niche filtering, though stochastic assembly processes likely play a role as well. These results indicate that sediment microbial communities are highly complex and sensitive to changes in land use practices.

## Introduction

Water-saturated sediments that underlie a stream channel (benthic and hyporheic zones), often harbor the majority of biomass in a riverine system, primarily in the form of microbial biofilms [1]. These sediment microbial communities dominate riverine biogeochemical cycling and can be responsible for 76–96% of total respiration [2]–[6]. Despite their importance, these microbial communities are poorly characterized [7], [8]. Mapping the spatial and temporal distribution of taxonomic and functional diversity in different lotic (river and stream) biomes, and understanding how this diversity is modulated by environmental and anthropogenic drivers, is vital for integrating microbes into predictive biogeochemical models [9].

Initial culture-independent studies investigating sediment microbial phylogenetic structure (e.g. PLFA, TRFLP, DGGE, clone libraries, etc.), have demonstrated that microbial communities are extremely sensitive to changes in the physicochemical state of freshwater sediments [10]–[13], and can be used as indicators of ecological degradation [14]. Although few metagenomes have been published from freshwater sediments [15], a handful of recent studies have examined water-column-associated microbial communities in river systems [16]–[18]. For example, signatures of allochthonous carbon mineralization were found in an Amazonia River metagenome [16], and novel functional genes have been identified in these underexplored environments [17], [18]. However, given the limited number of datasets and their spatial and temporal coverage, it is not yet possible to identify common themes governing the composition and functioning of freshwater communities, particularly those in river sediments.

Here we focus on the Tongue River, which flows out of the Big Horn Mountains of Wyoming and into southeastern Montana, past the Northern Cheyenne Indian Reservation, where it eventually empties into the Yellowstone River near Miles City, Montana. It passes through the geologically distinct Powder River Basin [19], which contains significant coal and natural gas deposits. Due to the geological heterogeneity and extensive fossil fuel extraction in this region, the Tongue River provides a useful model for understanding relationships between microbial taxonomic and functional diversity and physical geography and human land-use practices. Further, it allows us to test whether microbial community structure is shaped primarily by environmental selective factors or by stochastic fluctuations across space and time.

Here we examine freshwater sediment microbial diversity and function using replicated 16S rRNA amplicon sequencing (V4 region) and whole genome shotgun sequencing. We sampled six locations spanning 134 km of river for two years, both spring and fall seasons. Two main hypotheses were tested: first, that phylogenetic and functional beta diversity vary with specific environmental factors, rather than stochastically across space or time [20]; and second, that land use practices such as coalbed methane extraction and human settlements alter microbial community structure and function in stream sediments.

## Methods

### Sample Collection

**Figure 1:**
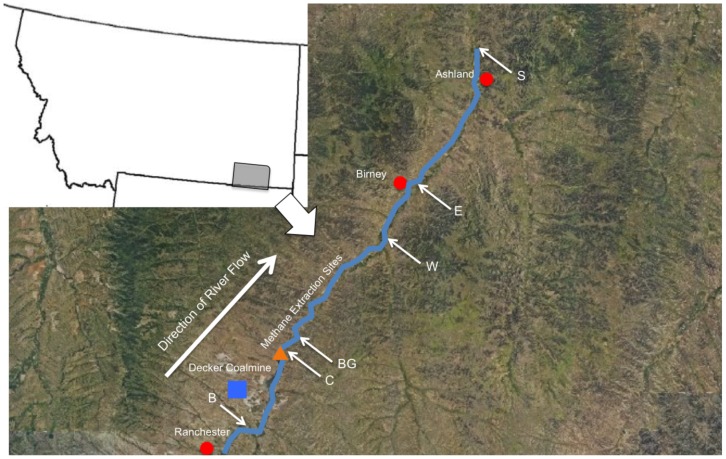
Location of sampling sites along the Tongue River. The upper left inset panel highlights the study region in southeastern Montana. The main panel shows a ∼140 km stretch of the Tongue River (blue line) that was sampled for this study. The direction of water flow is south to north. Notable features include the Tongue River Reservoir Dam (orange triangle), small towns (red dots), and the Decker Coalmine (blue square). The sites are described further in the text (Methods). The satellite image was obtained from the USGS Map Viewer website (http://viewer.nationalmap.gov/viewer/).

Six sampling locations (**Fig. 1** **; Table S1**) along ∼140 km of the river were sampled each Fall and Spring over a two year period (October 2010/2011 and March 2011/2012). Spring and fall time points were chosen for practical purposes (e.g. the river was frozen over in winter), and because these seasons are qualitatively different from one another (spring is characterized by snow-melt and flooding, while fall is characterized by lower flow rates and higher organic matter inputs). No specific permission was required for sampling these sites, and no endangered or protected species were involved in this study. Spatial distance (in kilometers) between sampling sites was estimated by tracing a path along the river using the ruler tool in Google Earth (http://www.google.com/earth/) (**Table S1**). All sites except B are downstream of the Tongue River Reservoir Dam. Sites B, E and S are each downstream of small towns (Ranchester, Birney, and Ashland, respectively). The B site is closest to the Decker Coalmine, and downstream from irrigated farmland. The C and BG sites are near coal bed methane extraction wells, and extraction water is piped directly into the river from adjacent wells. The W site is downstream of irrigated farmland.

Temperature, conductivity, salinity, and dissolved oxygen were measured *in situ* during sampling using a YSI85 probe (YSI Incorporated, Yellow Springs, OH) and pH was measured *in situ* using an UP-10 UltraBasic portable pH meter (Denver Instruments, Denver, CO). Sample sites were selected at riffles, where the water becomes shallow, with abundant fine-grained gravel. Two replicate sediment samples (∼15 g per sample) were collected from the top 20 cm of the streambed at each site, and sieved using sterile 2.36 and 1.76 mm sieves (stacked on top of one another), to obtain a homogeneous sediment particle size [14]. Samples were stored in sterile 50 mL centrifuge tubes on wet ice during transport for up to 6 hours and then stored at −80°C at Chief Dull Knife College (CDKC) prior to DNA extraction.

### DNA extraction and Sequencing

DNA was extracted using the PowerSoil kit (MoBio, California, USA), according to the manufacturer's instructions, at Chief Dull Knife College (Lame Deer, MT). Genomic DNA was stored at −20°C. Duplicate samples from all six sites and four time points, were used for amplicon sequencing (excluding sites BG and B for fall 2010, for a total of 44 amplicon samples; Supplemental Data File 1). Samples from the 2010/2011 sampling year with sufficient yield (at least 1 µg of gDNA) were also chosen for metagenomic sequencing (**Table S2;** 25 total metagenomes). Amplicon sequencing of the 16S rRNA gene V4 region was done using Earth Microbiome Project (EMP) standard protocols at Argonne National Laboratory (Argonne, IL), on the Illumina MiSeq platform (http://www.earthmicrobiome.org/emp-standard-protocols/). Shotgun metagenome library preparation and sequencing were carried out by HudsonAlpha (Huntsville, AL) on the Illumina HiSeq-2000 platform (100 bp paired end; insert size  = 150 bp). Metagenomic data is publically available on the MG-RAST server (MG-RAST IDs 4481969.3 - 4481980.3; **Table S2**). Amplicon data can be accessed in the EBI database under accession number ERP004510.

### Sequence Data Processing

#### Amplicon Data

All amplicon sequence analysis was performed using QIIME 1.6.0 [21]. A two-step open-reference operational taxonomic unit (OTU) picking workflow was used, as described in a previous study [22]. Briefly, reads were first clustered with a reference database, in this case the December 2012 Greengenes database [23] pre-clustered at 97% identity. Second, reads that did not group with any sequences in the reference collection were clustered *de novo*. Clustering at 97% identity was carried out using the UCLUST algorithm [24]. Representative sequences were chosen for each OTU (cluster centroids) and aligned against the Greengenes core set with PyNAST [25]. All OTUs whose representative sequences failed to align were discarded. A phylogenetic tree was built from this alignment using FastTree v2 [26], and taxonomy was assigned to each representative sequence, and by extension to the entire OTU cluster, using the RDP classifier [27] retrained on Greengenes. The number of sequences per sample (after default quality filtering and demultiplexing in QIIME) ranged from 55,444 to 88,272 (3,123,192 total). To obtain an equal number of sequences across samples, the amplicon OTU table was resampled to an even depth of 55,000 sequences per sample.

#### Shotgun Metagenome Data

Twenty-three samples were shotgun-sequenced to a depth of 15–23 million reads, and two samples were also sequenced to a higher depth (∼60 million reads; MG-RAST IDs 4481963.3 and 4481964.3). The two deeply sequenced samples allowed us to test how sequencing depth affected our results. Metagenomic data were quality-filtered and annotated through the MG-RAST pipeline [28], [29]. Processed metagenomic data were downloaded using the matR package (http://metagenomics.anl.gov) in R v2.15.2 [30]. For protein-coding genes, annotations were based on SEED Subsystems L3 [31]. The metagenome table contained 18,990,778 annotated reads, grouped into 474 functional categories. Functional annotations were rarefied to 311,760 hits (SEED Subsystems L3 annotations, - each representing an individual sequence) per sample, for all samples.

### Predicted Metabolic Turnover Analysis (PRMT)

A PRMT analysis [32] was used to evaluate the community metabolic potential between samples as a function of gene abundances. Enzyme commission (EC)-based gene abundances were extracted from the SEED Subsystems L3 annotations, as described previously [31]. Gene abundances were quantile-normalized and log_2_-transformed before analysis. The details of this method are given in [32]. Briefly, sample enzyme gene abundances are transformed by a weighted matrix of possible metabolic reactions (the environmental transformation matrix [ETM]) collected from the KEGG database (Ogata et al. 1999) as of September, 2010, and then compared to a ‘reference’ sample, which was also transformed by the ETM. This results in a set of metabolites with attributed PRMT scores for a given sample. Positive PRMT score values represent the consumption of a particular metabolite, and negative scores represent the accumulation of a particular metabolite. In this analysis, the ‘reference’ sample was an average of all samples, allowing PRMT scores to be directly compared across samples. Because these scores were calculated relative to the average metagenome, they cannot predict net production or consumption of each metabolite, only the relative flux [32]. To interpret the PRMT scores, metabolites were annotated back to KEGG functional pathways. For a given sample, PRMT scores from replicates were averaged. For a given KEGG pathway, the positive PRMT scores were summed to give a ‘net positive’ PRMT pathway value and the negative PRMT scores were summed to give a ‘net negative’ PRMT pathway value. The ‘net difference’ or ‘pathway flow’ was found by adding the ‘net positive’ and ‘net negative’ pathway values for each functional pathway in the sample. To test differences among sites, samples were grouped based on their location: “Coalbed” sites BG and W are downstream from coalbed extraction wells; “Smalltown” sites B, E, S are downstream of small towns; and “Tongue Reservoir” site C is downstream of the Tongue Reservoir. Groups’ ‘pathway flows’ were compared for a particular pathway using a Kruskal-Wallis test. Only significant (p<0.05) pathways are discussed.

### Statistical Analyses

All statistical analyses, dimensional reduction, and plot construction were preformed using QIIME v1.6.0 and R v2.15.2 [21], [30]. Beta diversity was estimated using the weighted UniFrac metric for 16S rRNA amplicon data [33] and the Hellinger transformation for shotgun metagenome datasets [34]. The Hellinger metric, unlike Bray-Curtis, minimizes the contribution of unshared factors (i.e. unique taxa/functions) to differentiating samples.

Non-parametric analysis of similarity (ANOSIM) was used to test for significant differences in community structure based on categorical variables, while Mantel tests (permutation-based) were used to test for significant correlations between community composition and numerical vectors. Mantel tests were performed using the Pearson product-moment correlation coefficient. BEST analysis (http://cc.oulu.fi/~jarioksa/softhelp/vegan/html/bioenv.html) was used to determine the optimal set of environmental variables for explaining variation in microbial community structure. Analysis of variance (ANOVA) was used to determine whether there were significant differences in OTU or gene abundances between samples or categories. Procrustes analysis was used to determine whether the distributions of two equivalent sets of samples based on amplicon and metagenome data were more similar than could be expected due to chance alone [35]. Further information on ANOSIM and BEST analyses can be found in the documentation for the *vegan* package in R (http://cran.r-project.org/web/packages/vegan/index.html).

Heatmap plots and hierarchical clustering were carried out using the heatmap.2 function from the gplot package in R (http://cran.r-project.org/web/packages/gplots/index.html).

## Results and Discussion

### Phylogenetic and Functional Diversity

In total, over all sites, 64,858 non-singleton OTUs (97% similarity cutoff) were identified from 3,137,798 reads. Strikingly, 93% of these OTUs are ‘novel’ (i.e. not found in the December 2012 Greengenes database [23]). Compared to other environments sampled by the Earth Microbiome Project (EMP) at an equivalent depth of sequencing (1,000,000 reads per sample), the average OTU richness in Tongue River sediments is approximately 48,000, while EMP marine sediment and soils – on average, the most diverse biomes studied by the EMP – have average richness values of ∼19,000 and ∼13,000, respectively (www.microbio.me/emp). Thus, the Tongue River is the most diverse ecosystem studied by the EMP to date. Moreover, the proportion of novel diversity is higher in Tongue River samples (93% of OTUs) than in any other environment analyzed by the EMP; by comparison, only ∼50% of marine sediment and ∼40% of soil OTUs are ‘novel’ (https://github.com/EarthMicrobiomeProject/emp). This preponderance of ‘novel’ diversity reflects the poor representation of freshwater sediment communities in existing sequence databases [36], [37].

In addition to unprecedented taxonomic diversity, Tongue River sediments also harbor extremely high functional gene diversity, as revealed by shotgun metagenomics. Metagenomic richness varied from 2,800–4,300 functional gene (SEED) annotations per sample, higher than the metagenomic diversity detected in a recent soil meta-analysis (1,200–2,500 functional gene annotations per sample) using comparable techniques [38]. While soils are often recognized as the most heterogeneous and diverse microbial ecosystems on the planet [39], recent work has revealed that sediments and microbial mats are much more diverse than previously thought [40]. Indeed, our results demonstrate that river sediment microbial diversity rivals or exceeds that of soil. As in soils, the high taxonomic and functional diversity of Tongue River sediments is likely due to the heterogeneous nature of the sediment environment.

### Community Structure Varies Along Temporal, Spatial, and Environmental Gradients

**Figure 2:**
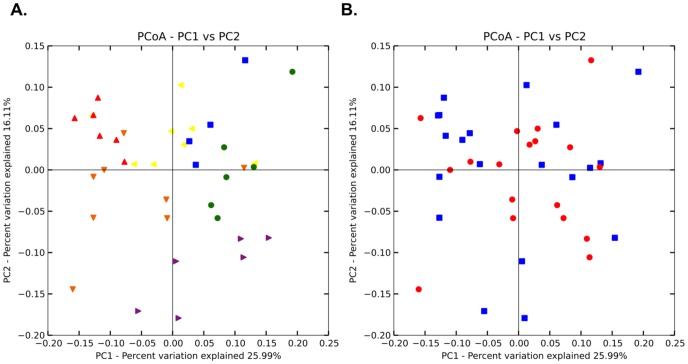
Principal coordinate plot (PCoA) of weighted UniFrac distances between Tongue River amplicon samples. Panel A is colored by sample location: sites B (blue), C (orange), BG (red), W (yellow), E (green), S (purple). Panel B is colored by season: fall (red) and spring (blue).

Average beta diversity distances were significantly smaller within a site than between sites (ANOVA, p<0.0001; **Fig. 2a**). BEST analysis [41] was used to construct the optimal multi-parameter model for the amplicon data (parameters included in the calculation: distance, temperature, DO, salinity, conductivity, and pH). After 1000 permutations, a two-parameter model produced the highest correlation statistic, with distance between sites and salinity as the most important variables (rho = 0.385). Individually, both salinity and distance were significantly correlated with community structure (Mantel-p <0.01). Spatial distance produced the optimal single-parameter model for explaining the variance in community structure (rho = 0.377), indicating that sites nearest in space were also most similar in community structure. When distance was removed from the analysis, salinity provided the most explanatory power as a single parameter (rho = 0.197). However, even when controlling for changes in salinity, spatial distance remained the strongest correlate with beta-diversity distance (partial Mantel, controlling for salinity: r = 0.29, p = 0.001). It is difficult to say how much of this correlation with distance represents neutral community assembly processes (e.g. dispersal limitation), and how much is confounded by qualitative (land use practices and geological heterogeneity) and quantitative (physical and chemical) site characteristics that differ along the river and often co-vary among adjacent sites. For example, adjacent sites E and S are both downstream from small towns, as is site B, at the opposite end of our transect. Sites W and E are adjacent and both downstream from irrigated farmland (as is site B); sites BG and C are both close to coalbed methane extraction wells. Future work to tease apart the contributions of spatial proximity and shared environmental characteristics will require intensive sampling of physicochemical parameters along the transect.

Although samples were collected in both Spring and Fall, season did not appear to be a strong driver of community structure. There was no significant difference between spring (5–13°C) and fall (6–16°C) temperatures (ANOVA p>0.1; **Table S3**). Likewise, there was no significant correlation between season and community structure (ANOSIM p>0.3; **Fig. 2b**; weighted UniFrac). However, community structure and time did show a significant relationship (i.e. collection date over the two-year sampling period; Mantel r = 0.14, p = 0.004), and this relationship may be driven in part by a significant positive correlation between salinity and time (Mantel r = 0.12, p = 0.03).

### Specific Taxa Vary with Environmental and Anthropogenic Drivers

**Figure 3:**
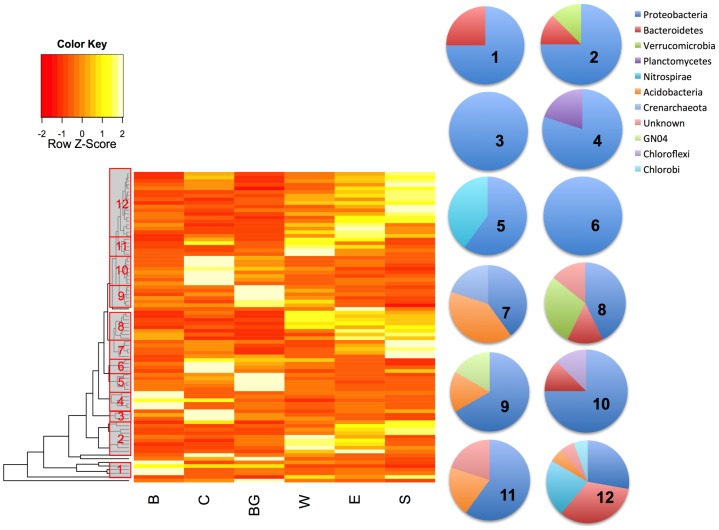
Relative abundance profiles of 85 abundant OTUs (more than 500 reads each) whose abundance varies by site (ANOVA, Bonferroni-corrected p<0.05). For each OTU, abundance across sites is normalized to give a mean of zero and a standard deviation of 1.0 (Z-scores denote the number of standard deviations from the mean in either direction). Dendrogram on the left represents the results of hierarchical clustering of OTUs. Grey boxes indicate clusters of OTUs with similar abundance patterns (identified as clusters #1–12). Pie charts show relative proportion of phyla within each cluster. Yellow represents higher normalized abundance and red represents lower normalized abundance.

A large number of individual taxa (337 OTUs) showed significant differences in abundance across sampling locations (ANOVA, Bonferroni-corrected p<0.05). Some of these OTUs were completely novel and could not be assigned even to phyla (n = 55, 16%). Of the remainder, 51% were *Proteobacteria*, 10.6% *Acidobacterium*, 6.7% *Bacteroidetes*, 6% *Planctomycetes*, 5% *Nitrospira*, 3.9% *Verrucomicrobia*, 3.5% *Chloroflexi*, and 3.1% candidate phylum WS3, along with several low-abundance phyla that together represented 10.2% of the OTUs. **Figure 3** shows the behavior of the 85 most abundant OTUs (≥500 reads) that differed significantly in abundance among sites. Several clusters of taxa showed markedly higher abundance at a single site: clusters 1 and 4 at site B, the furthest upstream, including particular members of *Methylophilaceae*, *Burkholderiales*, and *Sphingobacteriales*; clusters 3, 6, and 10 at site C, just downstream of the reservoir; and clusters 5 and 9 at site BG near the coalbed methane extraction wells, including members of the Betaproteobacteria and *Chromatiales* (purple sulfur bacteria). Overall, OTUs more abundant at the BG site fell into the *Proteobacteria*, *Acidobacteria*, *Nitrospirae*, and *GN04* phyla (**Fig. 3**). One large cluster of OTUs (cluster 12, Fig. 3) increased in abundance along the transect (upstream to downstream), peaking at the final site. This pattern mirrors an increase in salinity and human population density at the downstream sites and leads to the hypothesis that these taxa may be sensitive to these dual gradients. Finally, two OTUs from the *Crenarchaeaceae* family (within the *Thaumarchaeota*) were found in highest abundance at the E and S sites (varying as much as 13-fold across the sample sites), which are both downstream of small towns (Birney and Ashland, respectively; see cluster 7 in **Fig. 3**). The *Thaumarchaeota* include autotrophic ammonia oxidizers, which suggests potential ammonium oxidation downstream of Birney and Ashland. No individual OTUs were significantly correlated with DO or pH, and only four OTUs were significantly correlated with salinity (ANOVA, Bonferroni-corrected p<0.05). However, these results may be overly conservative, due to the multiple-test correction. Nonetheless, this suggests that dispersal and/or unmeasured environmental factors that vary across sites are stronger drivers of OTU abundance in this system than measured physicochemical parameters.

### Microbial Functional Potential Varies Along Environmental Gradients and with Land Use Practices

**Figure 4:**
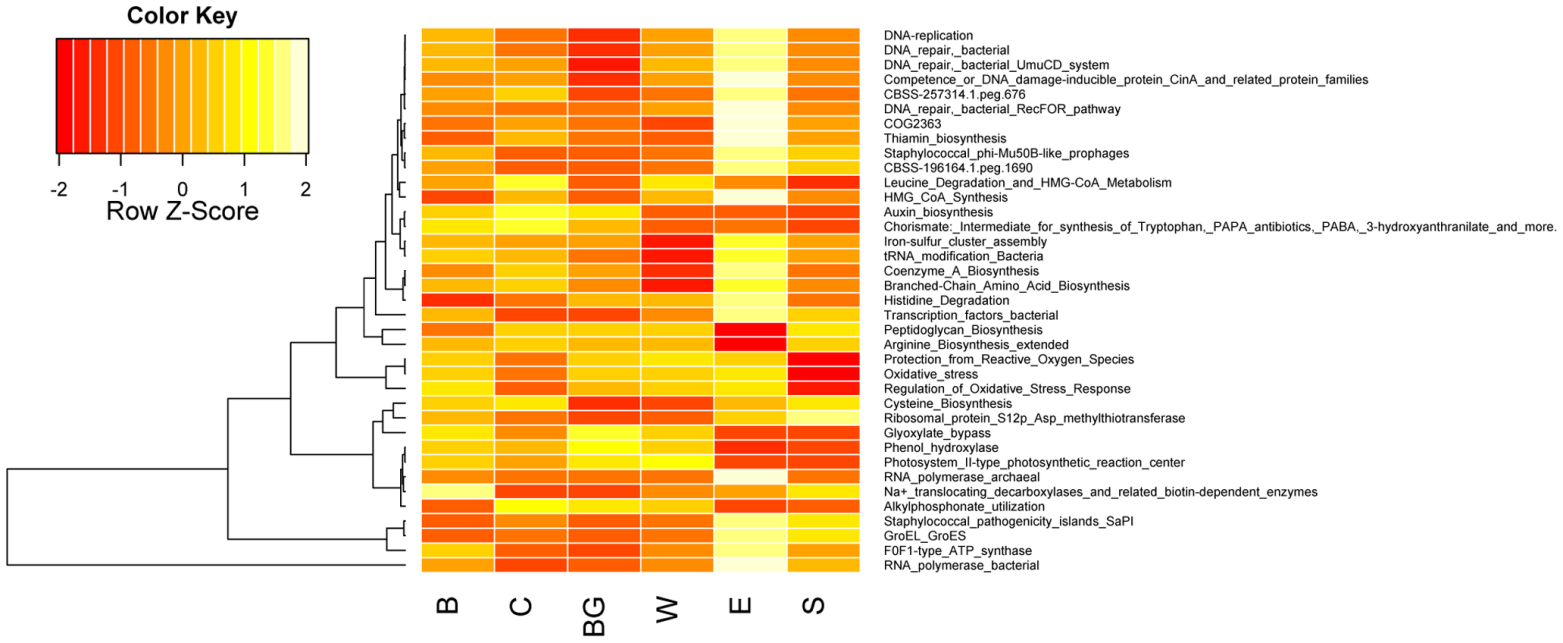
Relative abundance patterns of 37 functional groups (SEED Subsystems L3) that fluctuate significantly across sites (ANOVA, Bonferroni-corrected p<0.05). For each function, abundance across sites is normalized to give a mean of zero and a standard deviation of 1.0 (Z-scores denote the number of standard deviations from the mean in either direction). Dendrogram shows hierarchical clustering of functions based on profile similarity across sites. Yellow indicates higher abundance, while red represents lower abundance.

As with taxonomic composition, community functional gene composition was significantly correlated with distance (Mantel R = 0.34, p = 0.015), but not with season. Again, the correlation with spatial distance may be due to a combination of dispersal and environmental filtering processes. There were no individual functional categories (SEED Subsystems L3) that differed significantly between seasons. Across sites, however, there were 37 functional categories that showed significantly different abundances (ANOVA, Bonferroni-corrected p<0.05; **Fig. 4**). **Figure 4** shows the abundance patterns of functional genes that vary significantly across sample locations (ANOVA, Bonferroni-corrected p-value <0.05), grouped by hierarchical clustering. Most of the differences were due to the E site, which had higher abundances of over 20 functions, including oxidative stress tolerance genes (**Table S4**), DNA repair enzymes, and *Staphylococcus* phage genes and pathogenicity islands. The E site is located directly downstream of a small town and a saline tributary (Hanging Woman Creek), which may explain why it is so divergent from the other sites. There was a cluster of DNA repair and replication genes, along with a DNA competence pathway, which all exhibited similar behavior across sites (highest abundances at the E site; **Fig. 4**), which could suggest higher rates of horizontal gene transfer at the E site. Archaeal RNA polymerase was found to be most abundant at the E site, suggesting higher levels of archaeal abundance there (**Fig. 4**), consistent with the amplicon data. In addition to site E, sites B and S also showed enrichment in putative human-associated functions (staphylococcal prophage genes; SaPI) (ANOVA, Bonferroni-corrected p-value <0.05). This is not surprising, as B, E and S sites were all located downstream from human settlements. SaPI (pathogenicity island) genes behaved similarly to genes coding for bacterial transcription, ATP production, and chaperones at the E and S sites (**Fig. 4**), suggesting correlations with gene expression, energy metabolism, and stress response. Alkylphosphonate utilization genes were higher in abundance through the middle-stretch of the Tongue River (**Fig. 4**), downstream from coalbed methane extraction wells. This may reflect the fact that phosphonates are known constituents of a foam that is used to clear coal bed methane well pipes [42], and we observed the water from these wells being piped directly into the Tongue River. Ammonia assimilation potential was lower above the reservoir and was consistently higher at all sites downstream of the dam. This relative increase in ammonia assimilation genes was complemented by a significant decrease in nitrogen fixation genes (ANOVA, p<0.05) and a non-significant trend showing higher levels of nitrate utilization pathways and ammonification functional potential downstream of the reservoir.

If community structure and functional gene complements co-vary across space and time, it suggests that particular functions are coupled to particular taxa, indicating that there is phylogenetic conservatism of functional traits. To test this, we compared the core amplicon 16S rRNA community structure (only those taxa that were abundant [>200 reads] and present across all samples) and the functional gene structure (based on Hellinger distances calculated from abundance counts of SEED L3 annotations across samples) via Procrustes analysis. We used the core community amplicon data (which also reflects the most abundant taxa), rather than the total community, because our shotgun metagenomes only capture the most abundant taxa. Procrustes analysis revealed that patterns in the taxonomic structure and functional potential were significantly similar to one another (**Fig. S1**; M^2^ = 0.491, p<0.043), although there was a large average distance between equivalent points (indicated by a high the M^2^ value). The high M^2^ value suggests considerable functional variation at the strain level, in addition to large-scale phylogenetic conservatism. This result appears to be consistent with recent work based on whole genomes, showing that certain metabolic functions are strongly coupled to organismal phylogeny (e.g. oxygenic photosynthesis), while others functions seem to be almost completely decoupled from phylogeny (e.g. carbon substrate metabolism) [43].

**Figure 5:**
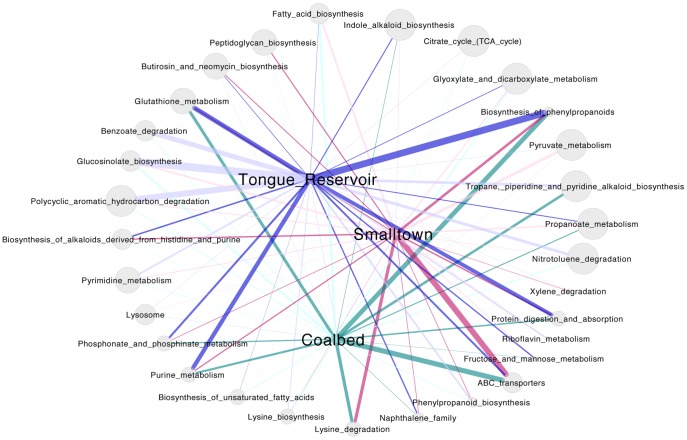
Net pathway flow analysis for Tongue River sediments. Nodes on the exterior (gray circles) are KEGG pathways that contain the metabolites predicted from PRMT analysis. Central nodes represent sample sites, grouped into Coalbed (sites BG, W; green), Smalltown (sites B,E,S; pink), and Tongue Reservoir (site C; blue). Edges between sites and pathways represent the average flow for that pathway across the given sites. Edge width is the magnitude of the flow; darker colors represent positive average flow, while lighter colors are negative average flow. Exterior pathway nodes are scaled according to their p-values (i.e. the significance of their deviation from the average metagenome), where larger nodes represent smaller p-values.

To further understand patterns in functional gene diversity along the transect, we used Predictive Relative Metabolic Turnover (PRMT), which predicts the relative turnover of metabolites based on functional gene abundances. This analysis generated 2205 predicted metabolites, associated with 264 KEGG pathways. For each pathway, ‘net pathway flow’ was predicted (see Methods), which is a function of the total consumption and production of all the metabolites in a given pathway, relative to the average metagenome (all samples pooled together). Net pathway flow values were then compared across groups of sites (‘Smalltown’, ‘Tongue Reservoir’, and ‘Coalbed’; **Fig. 5**). Thirty-three pathways had statistically significant differences in predicted net pathway flow (p≤0.05) between groups of sites. Net positive flow (i.e. metabolite consumption) was significantly higher for an antibiotic metabolism pathway, butirosin and neomycin biosynthesis, in the Smalltown group, relative to the other groups (mean net flow 1.66, compared with 1.16 and 0.15 for Tongue Reservoir and Coalbed groups, respectively; p = 0.018). This result implies that the metabolic potential for antibiotic metabolism was higher near small towns, which may be due to the anthropogenic release of antibiotics into the ground water. Two pathways of carbohydrate metabolism, glyoxylate and dicarboxylate metabolism and the citric acid (TCA) cycle, both had higher positive pathway flow in the Tongue Reservoir site compared to the other groups of sites (p<0.01) (**Fig. 5**), indicating higher relative flux through these pathways directly downstream of the reservoir. Pathways related to aromatic compound and hydrocarbon degradation also differed among sites, including Polycyclic aromatic hydrocarbon degradation, Nitrotoluene degradation, Naphthalene family, and Xylene degradation. In particular, Polycyclic aromatic hydrocarbon degradation showed much higher relative fluxes in the Smalltown and Coalbed groups than in the Tongue Reservoir site. These model results suggest that hydrocarbons are traveling downstream of the Decker coalmine, where they are being metabolized by the microbial community. Similarly, build-up of coal-tar associated metabolites [44], [45] was predicted at the Tongue Reservoir site, in close proximity to the Decker Coalmine, by pathway flow values for Nitrotoluene degradation and Benzoate degradation (mean net flow of −5.43 and −9.048, respectively), suggesting that coal substrates were present in the river.

### The Core Community and Wider Community Exhibit Similar Ecological Patterns

The core microbial community – OTUs present in all samples – consisted of 434 OTUs, which accounted for 49.9% of the reads in the original data set. As shown previously for marine ecosystems [22], these core OTUs were also all abundant (>200 reads), suggesting that our ability to detect even more core community members will increase with deeper sequencing. Moreover, the community structures of the core taxa and of the full dataset were highly correlated (**Fig. S2**; Procrustes analysis M^2^ = 0.037, p<0.0001), which would not be expected if beta-diversity patterns were driven by endemic taxa at particular sites along the river. This result suggests that these sediments are similar enough in environmental parameter space to select for a consistent set of taxa. Further, because of the large amount of rare diversity present in this system, it is unlikely that this core set of OTUs is assembled by stochastic processes, as they are consistently selected for across the transect, which suggests niche filtering. The most abundant OTU (also a core taxon shared across all sites) was classified in the order Rhodocyclales, which contains many metabolically versatile taxa that are often involved in xenobiotic degradation or alkane metabolism; organisms from this order are commonly used for remediation of contaminated substrates [46]. This organism was the most abundant taxon at all sampling locations, but its abundance was highest at the upstream sites (B, C, and BG), peaking in site C. Sites B, C, and BG are the most proximate sites to the Decker Coalmine.

There were 95 functional groups (SEED Subsystems L3) present in all the metagenome samples, together comprising the core metagenome. Most of the abundant functions were the same between the core and full data sets (**Fig. S3**). This result is consistent with the amplicon analysis, and indicates that, in addition to phylogenetic structure, the differences in functional potential between sites are predominantly due to changes in the relative abundances of a shared set of core functions. Together, the core community analyses show that changes in the phylogenetic and functional composition of these microbial communities are driven predominantly by ubiquitous taxa, and that ‘endemic’ microbes – present at a subset of sites – have little influence in determining the overall community structure.

### Conclusions

Given the sensitivity of sediment microbial communities to changes in physicochemical parameters, we hypothesized a strong dependence of microbial community structure and function on environmental parameters – i.e. environmental niche filtering. In support of this hypothesis, we found a significant, albeit weak, coupling between phylogeny and functional potential and a consistent core community of abundant taxa, suggesting that the distribution of particular taxa is driven by their metabolic capabilities in response to environmental drivers. However, the large amount of unexplained variance in the phylogeny/function correlation, along with significant correlations between distance and community composition, both hint at a role for neutral processes in structuring these sediment communities. In order to better understand the relative contributions of stochastic and deterministic processes in microbial community assembly in this system, a more controlled experiment with measurements of a larger diversity of environmental factors will be necessary. As for our second hypothesis – that land-use practices are an important driver of sediment microbial communities – we indeed found evidence for human impacts on community structure and function in stream sediments. Genes associated with pathogens (e.g. *Staphylococcus* phage genes) were more prevalent downstream from towns, and PRMT analysis predicted positive pathway flow for antibiotic metabolism in these sites as well. Moreover, we found evidence that resource extraction activities influence microbial community functional potential, with larger numbers of genes involved in phosphonate metabolism near coalbed methane extraction wells and a build-up of hydrocarbon-associated metabolites downstream of the Decker Coalmine.

Overall, we identified several environmental and anthropogenic drivers that help shape lotic sediment microbial community structure and functional potential. Our results suggest that both deterministic and stochastic forces are important for community assembly, and that beta diversity differences between sites are predominantly due to changes in the relative abundances of a shared core community. While much work remains to be done to assess the drivers of freshwater community structure and function, this work demonstrates the utility of metagenomic and amplicon sequencing for understanding human impacts on freshwater ecosystems.

## Supporting Information

Figure S1 **Procrustes analysis, comparing 16S rRNA-based community structure to functional gene community structure.** Each circle represents either the taxonomic or functional dataset; lines connect the two points for each sample. Colors: B.spring (red); C.fall (orange); C.spring (green) BG.spring (blue); W.spring (pink); W.fall (aquamarine); E.spring (purple), S.spring (yellow).
(TIF)

Figure S2 **PCoA of the core Tongue River community overlaid with PCoA of the full data set (amplicon data).** Equivalent samples are connected by a black edge, which denotes the distance between these points in the transformed coordinate space. Points are colored by site: B (blue), C (orange), BG (red), W (light blue), E (green), S (yellow), and pooled data across all sites (purple).
(TIF)

Figure S3 **The top 30 most abundant functional groups in the combined (all data) and core (only functions that are shared across all sites) metagenomes.**
(TIF)

Table S1 **Sampling location coordinates, and distances between sites along the path of the river.**
(XLSX)

Table S2 **Metagenomes sequenced for this study, showing MG-RAST identifier, site, season, and replicate information.** The bold MG-RAST IDs show the deeply sequenced samples (∼60 million reads per sample; compared to ∼20 million sequences for the other samples).
(XLSX)

Table S3 **Environmental metadata for Tongue River samples.**
(XLSX)

Table S4 **Average relative abundance of 37 functional groups (SEED Subsystems L3) that differed significantly between sites.**
(XLSX)
