# Supplementary figures and images for: Human and Environmental Impacts on River Sediment Microbial Communities
Source: PLoS One. 2014 May 19;9(5):e97435. doi: 10.1371/journal.pone.0097435 (PMC4026135; doi:10.1371/journal.pone.0097435)

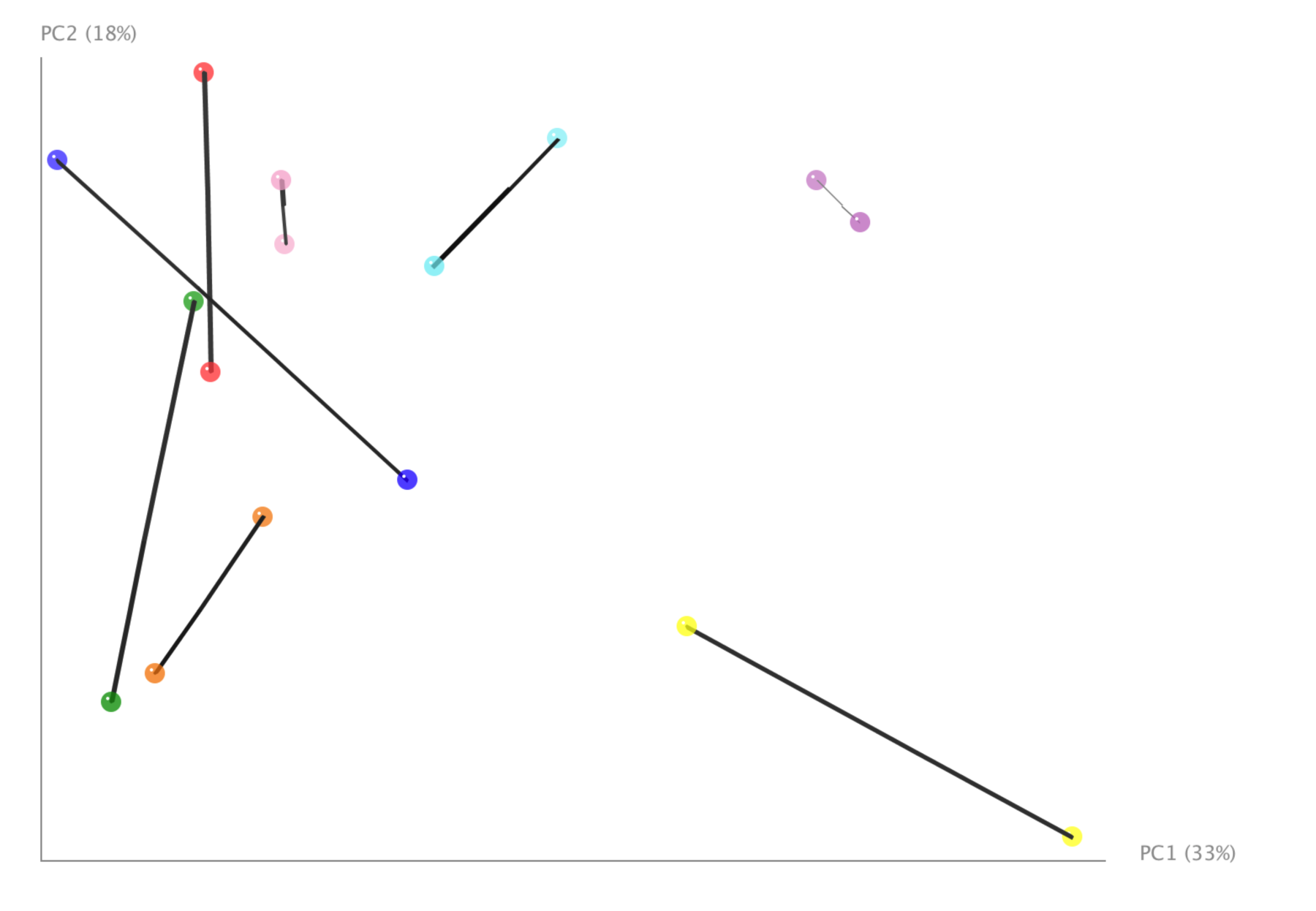

Supplement: Figure S1 — Procrustes analysis, comparing 16S rRNA-based community structure to functional gene community structure. Each circle represents either the taxonomic or functional dataset; lines connect the two points for each sample. Colors: B.spring (red); C.fall (orange); C.spring (green) BG.spring (blue); W.spring (pink); W.fall (aquamarine); E.spring (purple), S.spring (yellow). (TIF) [file pone.0097435.s001.tif]

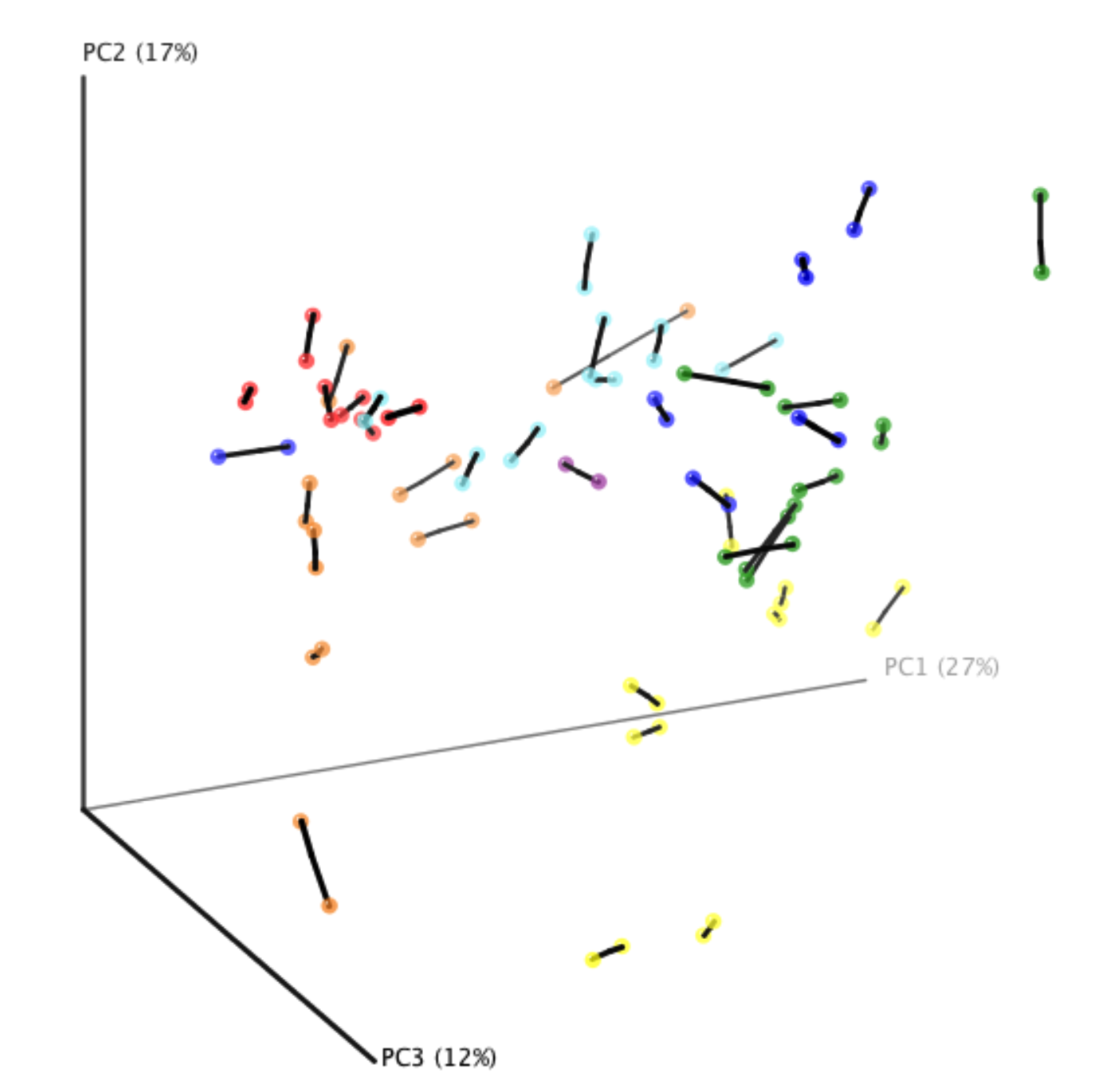

Supplement: Figure S2 — PCoA of the core Tongue River community overlaid with PCoA of the full data set (amplicon data). Equivalent samples are connected by a black edge, which denotes the distance between these points in the transformed coordinate space. Points are colored by site: B (blue), C (orange), BG (red), W (light blue), E (green), S (yellow), and pooled data across all sites (purple). (TIF) [file pone.0097435.s002.tif]

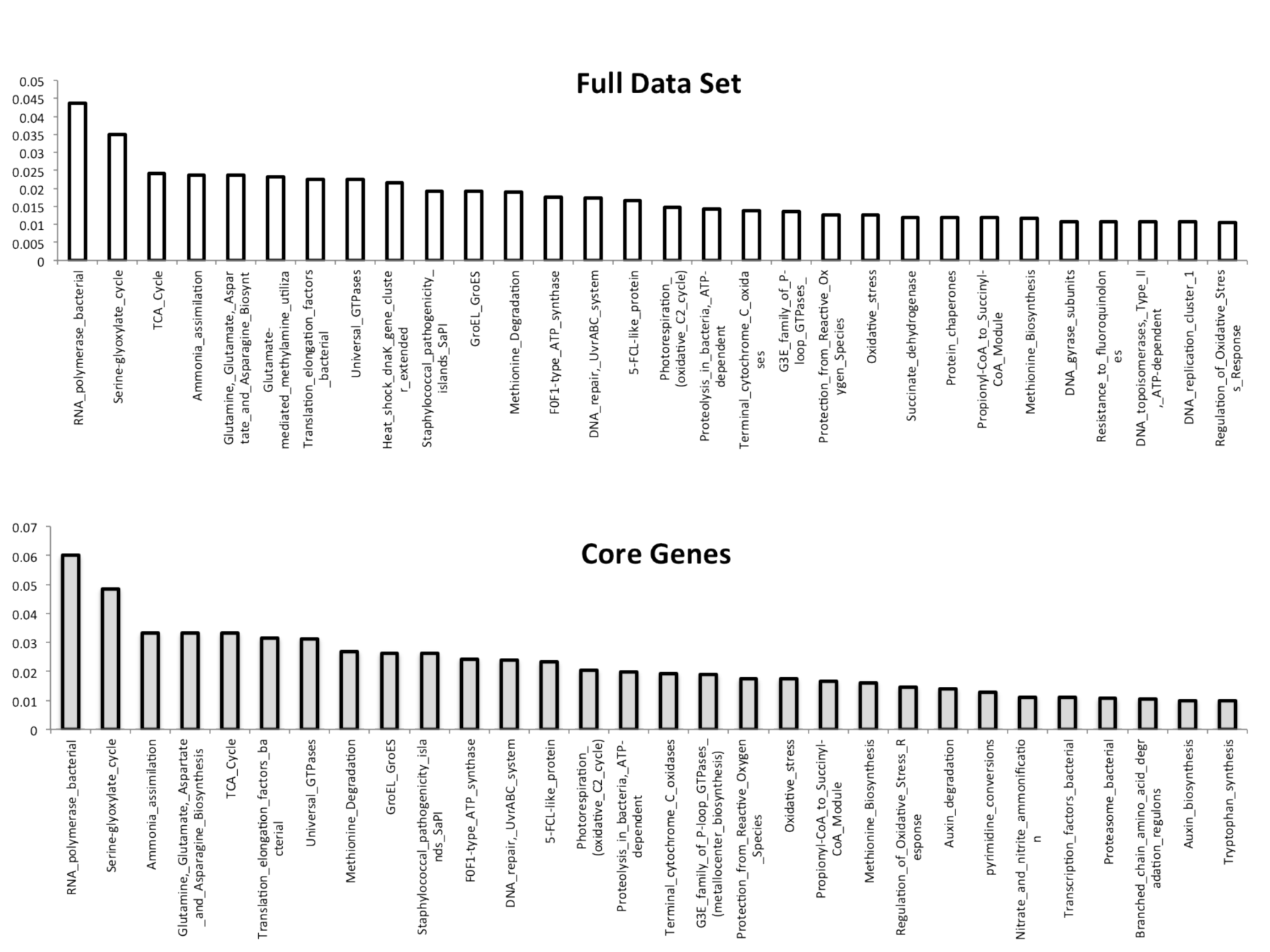

Supplement: Figure S3 — The top 30 most abundant functional groups in the combined (all data) and core (only functions that are shared across all sites) metagenomes. (TIF) [file pone.0097435.s003.tif]
